# Potential of Compost-Derived *Actinomycetes* for Low-Density Polyethylene Degradation

**DOI:** 10.3390/polym17172318

**Published:** 2025-08-27

**Authors:** Elżbieta Szczyrba, Tetiana Pokynbroda, Agnieszka Gąszczak, Nataliia Koretska, Stepan Tistechok, Ivan Roman, Oleksandr Gromyko

**Affiliations:** 1 Institute of Chemical Engineering, Polish Academy of Sciences, Bałtycka 5, 44-100 Gliwice, Poland; gaszczak@iich.gliwice.pl; 2 Department of Physical Chemistry of Fossil Fuels of the Institute of Physical-Organic Chemistry and Coal Chemistry named after L. M. Lytvynenko of the National Academy of Sciences of Ukraine, Naukova Str., 79060 Lviv, Ukraine; pokynbroda@ukr.net (T.P.); natalya.koretska@gmail.com (N.K.); 3 Genetic and Biotechnology Department, Ivan Franko National University of Lviv, 79000 Lviv, Ukraine; stepan.tistechok@lnu.edu.ua (S.T.); ivan.roman@lnu.edu.ua (I.R.); 4 The Culture Collection of Microorganisms—Producers of Antibiotics, Ivan Franko National University of Lviv, 79000 Lviv, Ukraine; oleksandr.gromyko@lnu.edu.ua

**Keywords:** biodegradation, low-density polyethylene, pretreatment, *Actinomycetes*

## Abstract

The growing concern over the long-term persistence of plastic waste has driven research into biological methods of breaking down polymers. This study investigated a process that combines physicochemical pretreatment and biodegradation of low-density polyethylene (LDPE) using bacterial strains isolated from commercial compost. Four bacterial strains were genetically identified and classified as *Actinomycetes*. Exposure of LDPE to these selected strains resulted in a measurable reduction in polymer sample weight, accompanied by alterations in surface hydrophobicity. Furthermore, the chemical modifications at the films’ surfaces were confirmed by the spectra obtained by Fourier transform infrared spectroscopy (FTIR). The microbial colonisation of plastic surfaces plays a key role in the overall biodegradation process. The formation of a biofilm and the subsequent morphological changes on the LDPE surface were revealed by scanning electron microscopy (SEM). The modification of the polyethylene surface by nitric acid treatment was found to be a promising strategy for enhancing the LDPE degradation. The acid-treated films exhibited the greatest weight loss, the greatest increase in carbonyl index values, and the greatest change in hydrophobicity following microbial exposure. Moreover, it was found that biodegradation under these conditions resulted in the lowest levels of phytotoxic byproducts. The transformation of polyethylene surface properties—from hydrophobic to hydrophilic—combined with the presence of oxidized functional groups made it easier for microorganisms to degrade LDPE.

## 1. Introduction

Plastics are a diverse family of materials varying in chemical composition and production methods. They consist of long-chain polymers derived from various sources, including natural gas, oil, and coal. Approximately twenty commonly used polymers exist, with polyethylene (PE) accounting for 26.3% of global production [1].

Due to its semi-crystalline structure, this polyolefin is considered to be highly persistent and has the potential to bioaccumulate, leading to various environmental and toxicological consequences. PE polymers have different densities and three-dimensional structures due to different manufacturing processes and the resulting different arrangements of the linear chains (LMWPE, LLDPE, LDPE, HDPE). Low-density polyethylene is most commonly used in the manufacture of single-use items and is therefore the most common type of plastic waste in landfills (69.13%) [2,3]. The widespread use of plastics has increased the comfort, safety, and hygiene of life, as these materials are extremely functional (e.g., lightweight, durable, and chemically resistant). However, it should be noted that these same characteristics are simultaneously responsible for the negative environmental impacts associated with their use. Plastic pollution is one of the biggest challenges of our time, as only 12% of plastic waste is incinerated worldwide, 9% is recycled, and the rest ends up in legal and illegal landfills. Managing and disposing of plastic waste without creating secondary pollution is one of the most urgent problems that needs to be solved in the shortest possible time [4].

The priority strategies employed in plastic waste management are material recycling, followed by chemical recycling processes such as pyrolysis. However, these technologies are not universally applicable, particularly in sectors like agriculture. Agricultural plastic waste often contains soil particles, plant residues, and agrochemical contaminants, which significantly reduce recycling efficiency and limit the feasibility of conventional recovery methods [5]. As a result, increasing attention is being directed toward alternative approaches, among which biodegradation, in its broadest sense, represents a particularly promising solution [6]. Biodegradation’s ability to decompose microplastics is another significant advantage. These are difficult to collect and separate, which makes them virtually impossible to recycle. Undoubtedly, the main advantage of biodegradation is the fact that it is a low-energy process carried out under mild conditions; microorganisms operate at ambient temperature and pressure. Microbiological systems do not require the use of solvents, which are typical of many chemical processes. This reduces both operating costs and environmental risks [7]. Furthermore, microorganisms can be genetically modified to enhance their effectiveness. In addition, they can be used directly in the contaminated environment, making this approach suitable for in situ remediation. These advantages make microbial degradation a sustainable and environmentally friendly alternative to conventional physico-chemical methods of LDPE waste treatment.

Polymeric materials can undergo biodegradation through the formation of biofilm and the chemical activity of microorganisms [8]. The initial stages of this process are biodeterioration and biofragmentation, during which the surface structure of the plastic is affected by the combined action of abiotic factors and extracellular enzymes. The purpose of this action is to enable microorganisms to adhere to the surface and form a biofilm. The adhesion of bacteria to surfaces is determined by numerous important factors, including the population of inoculated bacteria and their individual characteristics (e.g., cell wall components, motility), nutrient availability, and substrate surface properties (surface charge density, wettability, roughness, stiffness, and surface topography) [9,10].

Different species of bacteria release different types of enzymes that have different effects and mechanisms for degrading LDPE. The process of depolymerisation, most commonly catalysed by peroxidases or laccases, leads to the formation of oligomers, dimers, and monomers [11]. Thanks to specific cellular transport mechanisms, low-molecular-weight compounds—typically containing fewer than 50 carbon atoms—are able to penetrate the cytoplasmic membranes and enter the cell, where they undergo further metabolic processing [12]. This process is called bio-assimilation, which is then followed by mineralisation, defined as the intracellular conversion of hydrolysis products into microbial biomass, accompanied by the release of carbon dioxide and water [13,14,15].

The persistent presence of LDPE and other synthetic polymers in natural environments has led to their recognition as emergent substrates for diverse microbial taxa. As a result, numerous microorganisms have evolved the metabolic capacity to utilise polymers as carbon and energy sources. Microbial communities demonstrate the potential to successfully overcome various environmental challenges by adapting their metabolic processes to the changing conditions [16,17]. More than 20 bacterial species, including *Stenotrophomonas*, *Bacillus*, *Pseudomonas*, *Nesiotobacter*, *Staphylococcus*, *Achromobacter*, *Micrococcus*, *Comamonas*, *Acinetobacter*, *Brevibacillus*, *Rhodococcus*, *Serratia*, *Streptococcus*, *Streptomyces*, *Aspergillus*, *Ralstonia* and *Klebsiella*, have been documented to effectively break down various forms of polyethylene [18,19,20,21]. The literature indicates that the source of plastic-degrading microorganisms can be any ecosystem in which plastic contamination is present. Polyethylene-degrading bacteria have been found in a wide range of environments, including landfills, soil, and marine ecosystems, confirming the widespread potential of bacteria to assimilate synthetic polymer contaminants [22,23,24,25].

The biodegradation of plastic materials is influenced by several factors, encompassing not only environmental conditions such as temperature, pH, oxygen availability, ultraviolet (UV) radiation, and moisture, but also the chemical and physical properties of the polymer itself [3]. Key determinants include the surface hydrophobicity, the molecular weight and composition of the polymer, the degree of crystallinity, and the physical form of the material (e.g., film, pellet, powder, or fiber) [26,27]. As previously stated, the process of microbial degradation of plastics is closely associated with the biofilm formation on the material’s surface, and the above-mentioned factors have been identified as having a significant influence on this phenomenon. One promising strategy to enhance the biodegradability of polyethylene involves applying various pretreatment methods aimed at increasing the polymer’s susceptibility to microbial or enzymatic activity [26]. These activities result in a reduction in the polymer chain length, the introduction of polar functional groups into the matrix, and alterations in the surface morphology and crystallinity of the polymer [28]. Common pretreatment methods include thermal, oxidative, chemical, and mechanical treatments. Thermal treatment involves subjecting the plastic to elevated to induce structural modifications. Oxidative treatments, often utilizing UV radiation or exposure to oxygen-rich environments, promote the formation of functional groups on the plastic surface. Chemical treatments typically use agents such as acids, bases, or solvents to break down polymer chains or modify the surface properties. Mechanical treatments, like grinding or milling, increase the surface area of the plastic, thereby enhancing microbial colonisation and subsequent degradation [26].

The effectiveness of biodegradation depends primarily on the specific characteristics of the microorganisms and the environmental conditions in which they occur, as well as on the physical and chemical properties of the polyethylene and the pretreatment measures used. The studies presented in the current work investigate novel bacterial strains and their responses to the applied pretreatment methods, thereby contributing to the broader search for efficient microorganisms and techniques to enhance the microbial degradation of plastics.

## 2. Materials and Methods

### 2.1. Origin of Bacteria

Novel bacterial strains were isolated from the commercial compost bought from a local gardening store. LDPE film was buried in commercial compost for 10 months, after which it was retrieved and rinsed with sterile water. The isolation of bacteria was conducted through serial dilution and subsequent spread-plating on agar containing 0.1% LDPE powder (obtained from Thermo Fisher Scientific, Cambridge, UK) as the carbon source. Plates were incubated at 30 °C, and morphologically distinct colonies were purified and morphologically and biochemically characterised. The process of bacteria isolation was previously described by Szczyrba et al. [29]. A schematic diagram of the research workflow is provided in the Supplementary Materials to illustrate the experimental stages described in this study.

### 2.2. PCR Amplification and Sequencing of the 16S rRNA Genes

The actinomycete-like strains for the total DNA extraction were cultivated in TSB medium for 3–5 days at a temperature of 28 °C and a shaking rate of 180 rpm. The total DNA extraction procedure utilised in this study has previously been described [30].

The 16S rRNA gene was amplified by means of a polymerase chain reaction (PCR) using the universal primers 8F (5′-AGAGTTTGATYMTGGCTCAG-3′) and 1510R (5′-TACGGYTACCTTGTTACGACTT-3′). In a total volume of 50 μL, the PCR mixture consisted of 5 μL of 10× PCR buffer, 1.0 μL of deoxynucleoside triphosphates (10.0 mmol/L each), 0.5 μL of each primer (100 pmol/L), 0.5 μL of DNA polymerase (1 U/μL), 2.5 μL of dimethyl sulfoxide, 2.0 μL of DNA template, and 38.0 μL of Milli-Q-grade water. The PCR parameters were as follows: initial denaturation at 95 °C for 5 min, followed by 30 cycles of denaturation at 95 °C for 30 s, annealing of the primers at 53 °C for 30 s, extension at 72 °C for 90 s, and a final extension at 72 °C for 10 min. The PCR products were visualised on 1% agarose gel. A QIAquick Gel Extraction Kit (Qiagen, Venlo, The Netherlands) was used for the PCR products’ purification. The sequencing of the purified samples (5 μL) was performed by ExploGen LLC (Lviv, Ukraine) in accordance with the manufacturer’s recommendations. The obtained 16S rRNA gene sequences were analysed using Geneious version 9.1.2 software [31]. The 16S rRNA gene sequences of the isolated actinomycetes strains K2, K4, K5 and K6 were deposited in the GenBank of the National Center for Biotechnology Information (NCBI) under accession numbers PQ084729, PQ084730, PQ084731 and PQ084732, respectively.

### 2.3. The Phylogenetic Analysis of Isolated Strains

A phylogenetic tree was constructed using the 16S rRNA gene sequences of the isolated *Actinomycetes* strains and their closest neighbours. The nearest related species to the 16S rRNA were identified based on the Basic Local Alignment Search Tool data available within the NCBI’s database (https://blast.ncbi.nlm.nih.gov/Blast.cgi, accessed on 30 May 2024) and then obtained from the GenBank. Moreover, the multiple sequence comparison within the Log-Expectation alignment tool was used to align the sequences [32]. The phylogenetic tree was constructed based on this alignment using the neighbour-joining method with 1000 bootstraps according to the Kimura two-parameter method [33]. This was performed using Molecular Evolutionary Genetics Analysis Version 11 software [34].

### 2.4. Preparation of LDPE

In order to enhance the biodegradability of LDPE foil, a series of pretreatment procedures were implemented. The LDPE film, purchased from a retail shop, was cut into smaller fragments measuring 3 cm × 3 cm. The polyethylene pieces were subjected to either a temperature of 80 °C or immersion in a solution of 50% HNO_3_ (Avantor, pure p. a.) or 30% H_2_O_2_ (Chempur czda) for 120 min. Thereafter, the film pieces were washed with 70% ethanol for 30 min, followed by three washes with sterile distilled water. Finally, the film pieces were dried at 60 °C for 24 h.

### 2.5. Biodegradation Tests

Six pieces of LDPE film (thickness 0.033 mm, Befaszczot, Bielsko-Biała, Poland) were weighed and placed in 500 mL Erlenmeyer flasks containing 200 mL of basic mineral medium. The composition of the mineral medium was described by Szczyrba et al. [29]. Then, one of the isolated strains was added to the flasks; the initial optical density (OD) was 0.2 at a wavelength of 550 nm. The cultures were then left to incubate for a period of 180 days at a temperature of 30 °C and a speed of 130 rpm. After this time, the LDPE pieces were pulled out of the culture. The films were washed 3 times with 70% ethanol and once with sterile distilled water, before being immersed for 4 h in 2% sodium dodecyl sulfate (ROTH) aqueous solution to remove biofilm and other debris. The samples were dried at 60 °C for 24 h.

#### 2.5.1. Determination of LDPE Degradation

##### Weight Loss

After a 24 h drying period, the film fragments were weighed using a moisture analyser (MAX 50/1/NH, Radwag, Radom, Poland). The percentage of weight loss was calculated by dividing the difference between the initial and final weights by the initial weight and then expressing the result as a percentage. In the calculation of weight loss in the LDPE tests following pretreatment, the initial weight was taken as the mass of polyethylene measured for each chemical or thermal treatment before exposure to bacterial strains.

In these studies, all measurements were performed in triplicate. Mean values and respective standard deviations are presented in the Supplementary Materials.

##### The Water Contact Angle

The water contact angle is defined as the angle formed by the tangent to the surface of a deposited droplet on a solid surface at the point of contact amongst three distinct phases: solid, liquid, and gas. A water contact angle measurement enables the evaluation of the hydrophobic or hydrophilic character of the tested surface. An increase in the hydrophilicity of the sample may be attributed to its biodegradation. It is generally accepted that the contact angle of hydrophilic materials is less than 90°, whereas hydrophobic materials have a contact angle greater than 90°. The water contact angles of LDPE were measured both before and after incubation with isolated strains. In the presented tests, deionized water was employed to measure the contact angle, and the study was conducted with the use of a video camera (JVC™ GZ-EX355 Everio, Yokohama, Japan). The water contact angle was determined at room temperature.

##### pH Change

The pH of the culture was measured at the beginning and end of the degradation period. At the end of the process, the culture suspension was centrifuged, and the pH of the cell-free supernatant was checked.

##### FTIR Analysis

The attenuated total reflection (ATR) method, which is a widely used technique for the study of surface layer structure, was employed. This method enables the characterisation of alterations in polymer structure resulting from the impact of various factors, including microbial activity [35]. Infrared spectroscopic studies of LDPE films were conducted within the wavenumber range of 4000 to 400 cm^−1^ using a diamond crystal (Nicolet 6700, Thermo Scientific, Cambridge, UK). Four types of LDPE samples were analysed (each one was evaluated at three different locations): untreated, thermally or chemically treated, untreated or pretreated and then soaked in water, and untreated or pretreated and then incubated with the respective bacteria. Every spectrum was baseline-corrected and normalized using OriginPro 2024b software.

The extent of polyethylene degradation can also be represented by the carbonyl index CI, which serves as an indicator of the chemical oxidation of polyethylene. The literature reports several methods for calculating this index. In general, the CI formula can be expressed as the ratio of the absorbance of the carbonyl and reference peaks, or the areas of these peaks, or the specified areas under the bands [36,37,38,39,40,41].

In this paper, CI was calculated from the following formula:

(1) $$CI=\frac{area\;under\;band\;1800-1600\;{cm}^{-1}}{area\;under\;band\;1500-1440\;{cm}^{-1}}$$

The range of 1600–1800 cm^−1^ is indicative of the carbonyl group, while the range of 1440–1500 cm^−1^ is indicative of the methyl group [36,37]. The area under the band was calculated through OriginPro 2024b software options using the peak analysis tool. To minimize the impact of manual error, baseline corrections were standardized to “y = 0”. The carbonyl indices were calculated based on the average spectra obtained from four spectra for different fragments of the film.

##### SEM Analysis

The alterations in the morphology of the surfaces of the LDPE films that had been incubated with isolated bacteria were examined by scanning electron microscopy (SEM) (Phenome Pure, Thermo Fisher Scientific). The films were removed from the bacterial suspensions and fixed overnight with 3% glutaraldehyde (Thermo Scientific) in 0.1 M phosphate buffer solution (pH 7.4) (Avantor, pure p. a). The LDPE was then washed with 0.1 M PBS and dehydrated in ethanol solutions of 50%, 70%, 80%, 90% and 96% (*v*/*v*), followed by two additional dehydration cycles in absolute ethanol. Prior to imaging, the films were subjected to an overnight drying process and then sputter-coated with gold.

### 2.6. Phytotoxicity

The assessment of the phytotoxicity of the cell-free extract obtained following the centrifugation of the tested strains’ suspension was conducted at the stage of seed germination. The seeds of *Triticum aestivum*, L., employed in the static tests, were obtained from a local supplier. Prior to the experimental phase, the viability of the seeds was evaluated and found to be guaranteed at 90%. The static test was based on the observation of root elongation and germination of wheat (*Triticum aestivum* L.) seeds. In each Petri dish, 10 seeds were placed on filter paper, and 4 mL of cell-free effluent from the cultures or water as the control sample was added. The seeds were cultivated in the darkness at ambient temperature for five days. Following the completion of this period, the number of seeds that had germinated was enumerated, and the lengths of the roots were measured. This evaluation was conducted on four independent replicates. The percentages of relative seed germination, relative root elongation, and germination index were subsequently calculated using the equations provided below [42,43,44].

(2) $$Relative\;seed\;germination=\frac{seeds\;germinated\;in\;the\;supernatant}{seeds\;germinated\;in\;the\;control}\cdot 100$$

(3) $$Relative\;root\;length=\frac{mean\;root\;length\;in\;the\;supernatant}{mean\;root\;length\;in\;the\;control}\cdot 100$$

(4) $$Germination\;Index=\frac{Realative\;seed\;germination\times Reative\;root\;elongation}{100}$$

## 3. Results

### 3.1. Phylogenetic Characterisation of the Actinomycetes Strains

**Figure 1:**
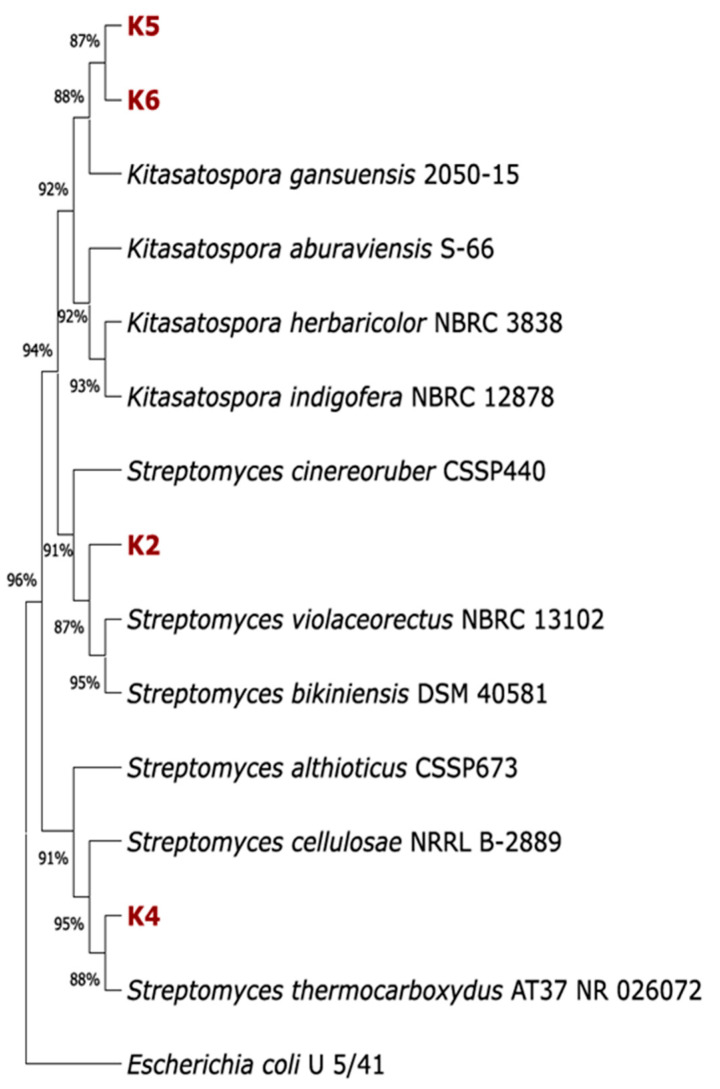
Phylogenetic tree of *Actinomycetes* isolated from compost based on the 16S rRNA sequences. A phylogenetic tree was constructed in MEGA 11 using the neighbour-joining method with 1000 bootstrap replicates. The 16S rRNA sequence of the *E. coli* strain U5/41 (NR_024570.1) was used as an outgroup.

The bacterial strains, designated K2, K4, K5 and K6, were isolated from compost purchased from a local garden shop. The protocol for their isolation was previously delineated in a separate publication [29]. The strains under investigation are characterised by typical actinomycetes-like growth. Analysis of the 16S rRNA gene allowed us to classify isolated strains as members of the *Streptomycetaceae* family. Strains K5 and K6 were identified as representatives of the genus *Kitasatospora* and formed a common clade with strain *Kitasatospora gansuensis* 2050-15, whereas strains K2 and K4 were grouped with members of the genus *Streptomyces*. Thus, strain K2 formed a clade related to the group of *Streptomyces violaceorectus* NBRC 13102 and *Streptomyces bikiniensis* DSM 40581, and strain K4 was grouped with *Streptomyces thermocarboxydus* AT37 (Figure 1).

*Actinomycetes* of the genus *Streptomyces* are widespread in soil and aquatic ecosystems. They have great potential as producers of antibiotics and other pharmaceuticals, but their role is not limited to this. *Streptomycetes* are capable of degrading complex biopolymers and are often used as degraders of synthetic pollutants. Members of the genus *Streptomyces* can degrade PET, PP, and LDPE [45]. The genus *Kitasatospora* is much less studied in this area. Currently, its ability to degrade chitosan [46] and resistance to heavy metals [47] are being studied. However, representatives of the genera *Streptomyces* and *Kitasatospora* are a promising source of new enzymes, including those responsible for polymer degradation.

### 3.2. Biodegradation Tests

#### 3.2.1. LDPE Film Weight Loss

A. Virgin films

Experiments conducted with recently isolated bacterial strains enabled the investigation of these microorganisms’ capacity to biodegrade low-density polyethylene. The dry weight loss of virgin LDPE films after 180 days of incubation was 1.86, 3.18, 0.93, 1.97% and 2.09% for isolates K2, K4, K5, K6, and consortium K2456, respectively. The observed reduction in weight can be attributed to the consumption of carbon from the LDPE film that was supplied to the culture. The negative control, which comprised an LDPE film devoid of biotreatment, exhibited no alteration in mass. The most effective strain was identified as K4, which caused the most significant loss of film weight.

Han et al. reported relatively limited LDPE biodegradation by Actinomycetes, with *Streptomyces* spp. achieving only 0.49% weight loss after 90 days [48]. In the present study, the compost-derived K strains exhibited markedly higher degradation rates, indicating a substantially greater capacity than that observed for many previously described Actinomycetes. Nevertheless, their performance remained below that of the most efficient bacterial strains reported to date, such as *Streptomyces coelicoflavus* and *Nocardiopsis alba*, which achieved 30% LDPE weight loss within 4 weeks and 32.25% within 30 days, respectively [49,50]. These comparisons highlight the pronounced strain-to-strain variability within Actinomycetes and underscore that, although not all representatives exhibit high biodegradation efficiency, certain isolates—including the K strains—possess considerable LDPE-degrading potential. Such strains merit further investigation as promising candidates for application in plastic waste bioremediation.

The results demonstrated in this work showed a similarity between K strains and *Kocuria palustris* M16, *Bacillus pumilus* M27, and *Bacillus subtilis* H1584, as previously described by Harshvardhan et al. [51]. Following a 90-day incubation period, the weight reduction was 1.4, 1.72, 1.26, and 0.97% for bacterial isolates H-237 (*Cobetia* sp.), H-255 (*Halomonas* sp.), H-256 (*Exiguobacterium* sp.) and H-265 (*Alcanivorax* sp.), respectively [50]. It is worth noting that a number of studies have been published that have demonstrated more efficient degradation of plastics than that which is described in this study. The discrepancy in the percentage weight loss compared to the existing literature can be attributed to several factors, including the thickness of the film used, the presence of modifying additives, and specific culture conditions (e.g., the length of incubation). The low-density polyethylene (LDPE) used in existing studies varies significantly. In the present study, a 33-micron-thick film was used, while Han’s et al. study employed a 6-micron-thick film [48], and Khandare’s study used a film only 0.2 microns thick [52]. Duddu et al. and Priyadahi used LDPE in powder form [49,50], whereas Harshvardhan used polyethylene bags. These differences in form and thickness may account for the observed variations in the polymer’s biodegradability [50].

B. Pretreatment

Oxidation is the main driver of polymer degradation and leads to many changes in the chemical, thermal, mechanical, and electrical properties of the material [38]. In order to enhance the biodegradation of LDPE by the isolated strains, a range of polymer pretreatment techniques were employed. The objective of these treatments was to reduce the average length of the chains present and to enhance the quantity of hydroxyl and carbonyl groups. The LDPE pieces were either subjected to a temperature of 80 °C or they were immersed in a solution of 50% HNO_3_ or 30% H_2_O_2_.

In the physical method of polymer treatment, high temperatures were applied to the LDPE film. Heat causes chemical bonds to break and free radicals, which are known to be extremely active, to form. These interact with surrounding molecules, breaking polymer chains and creating further free radicals [53]. The application of thermal pretreatment to LDPE resulted in enhanced biodegradation of the polymer in cultures comprising all isolated microorganisms. The K4 strain exhibited the greatest efficiency, reducing the mass of the LDPE material by 4.12%. Concurrently, it was observed that thermal pretreatment of the film resulted in an approximate twofold increase in LDPE biodegradation (within the K5 culture) in comparison to the untreated film.

The utilisation of heat treatment as a methodology to enhance the efficiency of plastic biodegradation has been referenced in the works of Balasubramanian and Arkatkar [54,55]. A threefold augmentation in weight loss of HDPE was observed during the biodegradation of this polymer by *Aspergillus terreus* MF13 [54]. In the study by Arkatkar et al., the degradation of polypropylene after heat treatment by bacteria was observed to be in the range of 0.4–0.7% [55]. Notably, this value was lower than that observed in experiments conducted in this study.

The application of hydrogen peroxide to the film did not result in a significant increase in the degree of LDPE biodegradation for the majority of the isolated strains. Furthermore, as demonstrated in Table 1, the degradation of the H_2_O_2_-treated film was considerably lower compared to LDPE without pretreatment for strains K2, K4, K6, and the bacterial consortium. The biodegradation process was significantly intensified only in the culture of strain K5, where a threefold increase in film degradation efficiency was observed.

Among the pretreatment methods employed, the introduction of carbonyl groups into the polymer backbone by nitric acid was found to be an effective approach through which to accelerate the degradation of polyethylene. In the cultures of all isolated bacteria, a significant increase in the mass loss of the polymer was observed for the film that had undergone pretreatment with a 50% nitric acid solution. After the biodegradation process, the highest mass loss was observed for isolates K4 and the consortium. However, the most significant decrease in the mass of nitric acid-treated LDPE compared to untreated polyethylene was observed for strain K5 and the consortium. In these cases, a more than twofold increase in weight loss was noted. As demonstrated by Rajandas et al., *Microbacterium paraoxydans* (GenBank ID: HQ185284) also exhibited a higher degradation rate of LDPE, following pretreatment of LDPE with nitric acid [56]. Therefore, the modification of the polymer backbone by nitric acid treatment was found to be a promising strategy for enhancing the degradation process of polyethylene.

#### 3.2.2. Water Contact Angle

Water contact angle (WCA) determination is a relatively simple method with the potential to yield meaningful data regarding the surface properties of a given material primarily on hydrophobicity/hydrophilicity. The WCA of untreated low-density polyethylene (LDPE) film is about 90–95°, making it hydrophobic. The water contact angle of a film can vary depending on the surface treatment or thickness of the film [57]. Hydrophobicity represents a significant determinant in bacterial adhesion and degradation processes. The hydrophobic nature of plastic requires bacteria to engage in hydrophobic interactions with its surface. Table 1 depicts the contact angles of pretreated films and post-microbial films for isolated strains and consortium. The value of the contact angle remained constant after abiotic treatment and was no greater than 90 degrees. Furthermore, the surface of the LDPE films from the cultures containing strain K5 or the bacterial consortium exhibited a greater degree of hydrophilicity in comparison to the surface of the control film. The reduction of contact angle occurred irrespective of whether LDPE was subjected to degradation without any pretreatment or after pretreatment. However, the most substantial alterations were observed in films which were subjected to nitric acid treatment prior to their introduction into microbial systems. The lowest contact angle was measured for the film after acid pretreatment and biotreatment with the K6 strain.

These results are in line with those of Han et al., who demonstrated that Streptomyces isolated from agricultural soils decreased the WCA of polyethylene films by 5.3%. These reductions in hydrophobicity are likely the result of biofilm formation and enzymatic surface oxidation typical of *Actinomycetes*. The contact angle of untreated plastic film, weathered plastic film, and buried plastic film exposed to *Arthrobacter* sp. and *Streptomyces* sp. decreased from 3 to 13%, depending on the strain and pretreatment type. Furthermore, the combined action of the two strains resulted in a significant decrease in contact angle, reaching a 6% to 9% reduction [48].

The contact angle of the low-density polyethylene film was measured following biological treatment with *A. nosocomialis,* and a decrease of 6% was found in comparison to the control film [58]. As demonstrated by Samantha et al. [59], a significant decrease in the contact angle of LDPE was observed following incubation in *Bacillus tropicus* MK318648 cultures. Prior to the application of microbiological treatment, the contact angle was measured at 98.7°. Subsequent to treatment with bacteria, a decline to 69.5° was recorded after a period of 40 days. In addition, the *Acinetobacter* LW-1 strain enhanced the hydrophilicity of LDPE by 16° [25].

#### 3.2.3. Changes in pH

In the process of microbiological degradation, pH is one of the parameters characterizing polymer degradation. The change in pH usually indicates the metabolic activity of a given strain. The pH of the mineral substrate was checked both before and after incubation with LDPE. The most significant decrease in pH value in systems containing untreated films was observed for strain K6 (Table 1). In the remaining cultures and abiotic systems, relatively small decreases in pH were noted.

This observation corresponds to findings by Duddu et al., where a decline in pH was recorded during Streptomyces-mediated LDPE degradation, presumably due to the accumulation of organic acids [49].

Changes in pH during polyethylene degradation were also observed by other researchers, e.g., Awasthi et al. [60] noted a decrease in pH from 7 to 5.7. These results indicate the metabolic activity of *Klebsiella* bacteria developing in the presence of heat-treated HDPE. In the process of polymer biodegradation by strains *Ralstonia* sp. SMK2 and *Bacillus* sp. SM1, pH decreased from 7.12 to 6.67 and from 7.12 to 7.03, respectively [18]. However, it should be noted that in the low-density polyethylene degradation process using microorganisms, a decrease in pH is not always observed. For instance, *Bacillus tropicus* MK318648 has been observed to induce an increase in the pH of the medium. The authors posited that this increase is attributable to the efficient production of extracellular alkaline protease by *Bacillus* species. Moreover, this bacterial strain was also found to lead to the release of ammonia in the presence of the tryptone component of the media, thereby resulting in an increase in the pH value [59].

#### 3.2.4. FTIR Analysis

The next stage of the research involved a comparative analysis of the FTIR spectra of both pretreated and untreated LDPE films following biodegradation. Alterations in the polymer’s functional groups after incubation with isolated bacterial strains were analyzed using Fourier-transform infrared spectroscopy with attenuated total reflection (ATR-FTIR). This technique was selected because the oxidation of LDPE by bacteria predominantly occurs on the polymer surface.

**Figure 2:**
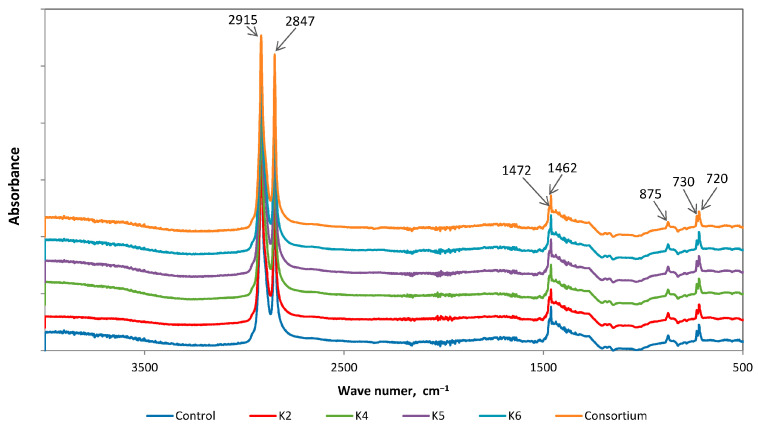
ATR-FTIR spectra of LDPE without pretreatment after biodegradation by bacterial strains isolated from commercial compost.

The characteristic absorbance bands of LDPE are located at 2915 cm^−1^ (strong), 2847 cm^−1^ (strong), 1462 cm^−1^ (medium), and 720 cm^−1^ (medium) (Figure 2). Additionally, weak peaks may appear at 1375 cm^−1^ and 1305 cm^−1^ [61].

**Figure 3:**
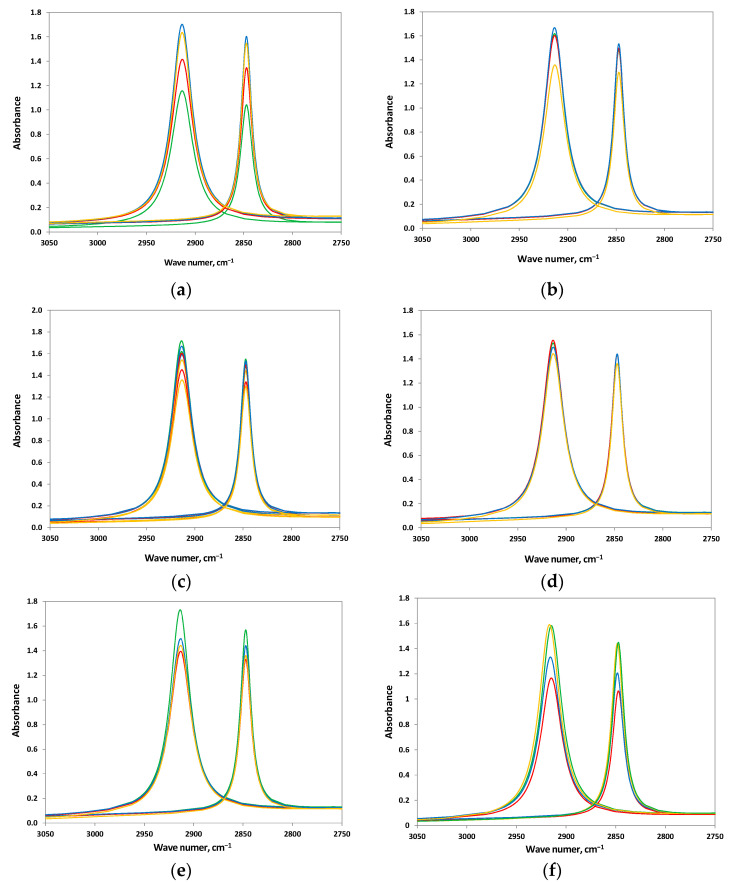
Comparison of changes of the 2915 cm^−1^ and 2847 cm^−1^ bands for pretreated LDPE after incubation with isolated strains. Green—LDPE without pretreatment; red—temperature pretreatment; blue—hydrogen peroxide pretreatment; yellow—nitric acid pretreatment. (**a**) K2; (**b**) K4; (**c**) K5; (**d**) K6; (**e**) consortium; and (**f**) without bacteria.

The study was conducted by comparing the differences determined from the FTIR spectra of films taken from bacterial cultures and control samples. Spectra analysis showed a decrease in band intensity at wavenumbers 2915 cm^−1^ and 2847 cm^−1^ for untreated films from cultures of K2 and K6 strains (Figure 3), which is the result of C-H bond cleavage [56,62,63]. Results of similar studies, i.e., subjecting polyethylene to biodegradation under aerobic conditions, can be found in the literature. They showed an increase, decrease, and no change in the intensity of the bands 2915 cm^−1^ and 2847 cm^−1^ [48]. A decline in these peak intensities was detected in experiments on LDPE films under the influence of bacterial isolates H-237, H-256, and H-265 [36,52] and LLDPE films exposed to *B. velezensis* MT9 [64]. In the study conducted by Samanta et al., no alterations were detected at 2915 cm^−1^, 2928 cm^−1^, and 2848 cm^−1^ during the depolymerisation process of LDPE in the presence of *Bacillus tropicus* MK318648 [59].

A detailed deconvolution of the spectrum in the range 3050–2750 cm^−1^ was performed to enable the complete separation of adjacent peaks (Figure 3). The most significant decrease in C-H band intensity for wavenumbers 2915 cm^−1^ and 2847 cm^−1^ (compared to films without pretreatment) was observed for films after heat pretreatment in K5 and constortium cultures. Pretreatment at a higher temperature increased the bioavailability of LDPE, thus facilitating its use as a carbon and energy source for the K5 strain. The use of nitric acid as a pretreatment method resulted in the most significant decrease in the intensity of the bands at 2915 cm^−1^ and 2847 cm^−1^ for LDPE exposed to strain K4. Among the pretreatment techniques employed in the present study, nitric acid treatment—through the incorporation of carbonyl groups into the polymer structure—proved particularly effective in promoting the degradation of polyethylene.

In addition, new peaks were detected in the 1650–1630 cm^−1^ region of these spectra, and a change in the shape of the peaks at 1472 cm^−1^ was observed. The occurrence of an increase in pre-existing peaks or the presence of new peaks (for example, indicative of hydroxyl (O-H), alkane (C=C), or ester (C-O-C) groups) suggests the formation of intermediates. This phenomenon was observed in the spectrum of polyethylene samples exposed to *P. plecoglossicida* SYp2123 [65]. Similarly, the treatment of LDPE with bacterial strains APCK5 and APCZ14 resulted in the emergence of novel peaks indicative of the formation of double bonds, acids, and ketones [66].

The process of oxidation has been proven to result in the reduction of the polymer’s molecular mass, as well as the formation of a number of oxygenated groups, with a particular prevalence of carbonyl groups [67]. The analysis of carbonyl formation by IR spectroscopy is the most widely used analytical method for assessing the oxidation of solid samples due to the strong absorption characteristics of the C=O stretching vibrations [68].

**Figure 4:**
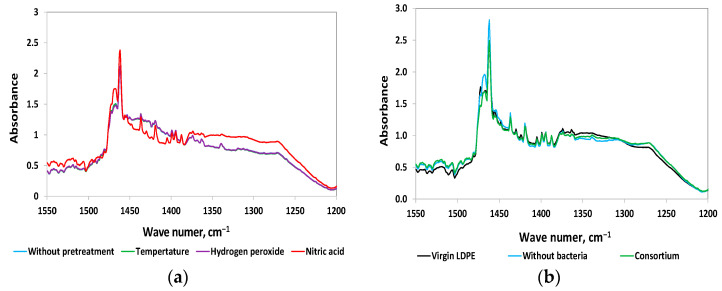
FTIR-ATR spectrum changes in the range of 1550–1200 cm^−1^ for pretreated LDPE samples: (**a**) changes observed after different pretreatments of LDPE followed by incubation with strain K6; (**b**) spectrum after nitric acid pretreatment of LDPE.

Upon comparison of the ATR-FTIR spectra of LDPE after incubation with K6 cultures, significant changes were observed in the 1550–1200 cm^−1^ region for samples pretreated with nitric acid, as opposed to those subjected to other pretreatments and the untreated controls (Figure 4a). After normalization, the spectra of the untreated foils and those pretreated with hydrogen peroxide or temperature overlapped. The spectrum of LDPE post nitric acid pretreatment showed a significantly altered spectral profile, with enhanced 1437 cm^−1^ and 1420 cm^−1^ bands. A similar effect was found for the remaining isolated bacterial strains and consortium.

To investigate the chemical modifications within this spectral range further, the following were compared: virgin LDPE; LDPE pretreated with nitric acid and then incubated in an abiotic mineral medium; and LDPE pretreated with nitric acid then incubated with a microbial consortium (Figure 4b). When analysing the ATR-FTIR spectrum of virgin LDPE, it was possible to observe the presence of peak 1472 cm^−1^, which was not detected after incubation in cultures with and without bacteria. Additionally, peak 1465 cm^−1^ appeared. These differences in the analysed ATR-FTIR spectra suggest that pretreatment with nitric acid induces significant structural and chemical changes in the LDPE material.

The carbonyl index (CI) is a key analytical parameter used to quantify specific alterations in the chemical structure of a polymer, particularly in relation to oxidative degradation. It serves as an indirect measure of the degree of polymer oxidation and is typically calculated as the ratio of the absorbance (or area) of the carbonyl functional group band to that of a reference band. The selection of an appropriate reference peak is critical and is based on the criterion that the chosen peak remains stable in both shape and intensity, irrespective of the changes occurring in the carbonyl region [36]. For polyethylene, a peak in the range 1500–1420 cm^−1^ seems to be the most suitable, but sometimes an alternative peak is chosen [69].

The calculation of the CI was performed using the FTIR spectra of the LDPE samples following an incubation period of 180 days with isolated strains. The use of various pretreatment methods and isolated bacterial strains resulted in an increase in the carbonyl index value in comparison to the CI of untreated or virgin films. The highest result was obtained for the polymer after nitric acid pretreatment and incubation in cultures with the bacterial consortium. An increase in the carbonyl index of LDPE was observed after both pretreatment and microbial incubation and suggests the oxidative degradation of the polymer. Chemical pretreatment leads to the formation of oxygen-containing functional groups, including carbonyls (e.g., ketones, aldehydes, and carboxylic acids). Subsequent to the process of incubation with bacterial strains, there is a continuation of the rise in the CI value, which is indicative of ongoing oxidative processes that are probably mediated by microbial enzymes, including oxidases.

**Figure 5:**
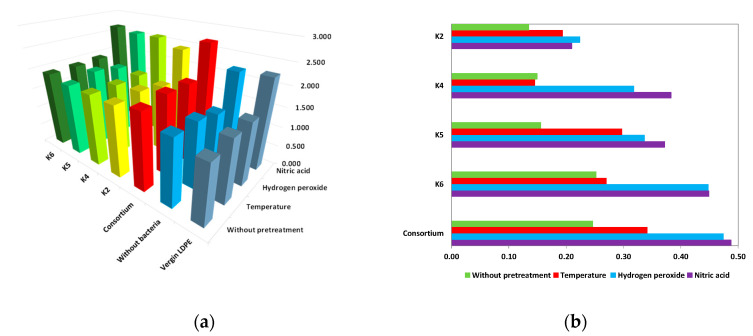
The carbonyl index (CI) of LDPE after biodegradation. (**a**) A comparison of the CIs of polymers after treatment with isolated bacterial strains. (**b**) Differences in the CI of LDPE after treatment with *Actinomycetes* and LDPE incubated without bacterial inoculation for various pretreatment variants.

The differences in the carbonyl index values of the film after incubation in bacterial cultures and the film after immersion in abiotic mineral salt solutions, for different pretreatment variants, as shown in Figure 5b, clearly confirm the impact of bacterial activity. The most substantial increases in CI were observed for LDPE samples taken from cultures inoculated with strain K6 or the consortium, particularly for films after hydrogen peroxide or nitric acid pretreatment. In contrast, the lowest CI variations were recorded for strain K2, where the applied pretreatment resulted in only minor modifications to the carbonyl index.

The appearance of changes in the spectral signal between wavenumbers 1600 and 1850 cm^−1^ for samples after microbial treatment indicates an ongoing biodegradation process. It should be noted that the intensity of the peaks in this wavenumber range does not remain constant but fluctuates during the process. It initially increases but decreases after prolonged exposure to microorganisms. This phenomenon can be attributed to the metabolism of carbonyl groups by microorganisms in a process termed beta-oxidation [70]. For all strains, the highest CI values were obtained for films after acid treatment. Relatively high CI values were calculated for the consortium for all four variants of film preparation.

In the majority of the samples’ spectra (with the exception of the spectra of the films treated with nitric acid), an additional peak appeared at wavenumber 875 cm^−1^, indicating the presence of calcium carbonate. CaCO_3_ is a filler widely employed to enhance mechanical and thermal properties while reducing production costs. The greatest benefits were observed after using CaCO_3_ at a concentration of 10% by weight [71,72].

Comparing CI values reported in publications is very difficult due to the different methods of preparing spectra (baseline correction, normalization, etc.) as well as the equations used (ratio between peaks heights, peaks areas or bands areas) [36,40,41]. In addition, in the case of biodegradation, the carbonyl index changes value during the course of the culture and is a time-dependent quantity.

Despite many years of using this method, there are still no clear rules for determining the carbonyl index [36,41]. Therefore, with so many different variants of CI measurement, it seems impossible to compare results. Furthermore, it has not been established what CI value corresponds to a significant degree of oxidation of the polyolefin material [38,73]. The carbonyl index is a reliable tool for comparing spectra of similar samples (material, shape, thickness) obtained with the same instrument.

Among the methods used for preliminary film treatment, immersion in a nitric acid solution had the greatest impact on weight loss, contact angle and pH changes, as well as on the increase in carbonyl index. This type of pretreatment proved particularly beneficial for films introduced into both the K4 strain culture and the consortium culture.

#### 3.2.5. SEM Analysis

The formation of a microbiological biofilm on the surface of the polymer is widely acknowledged to be a crucial step in the biodegradation process [48]. In the present study, pristine and pretreated LDPE films were inoculated with isolated bacterial strains in order to observe the development of biofilm on their surfaces. First, the development of biofilms was evaluated, and in the next step, the condition of the exposed surfaces following the removal of biofilms was examined. Many works highlight the correlation of the dense biofilm present on the polymers’ surface with their efficient degradation [74,75,76,77]. SEM analysis has proven to be a highly effective method for assessing biofilm development on the LDPE surfaces.

**Figure 6:**
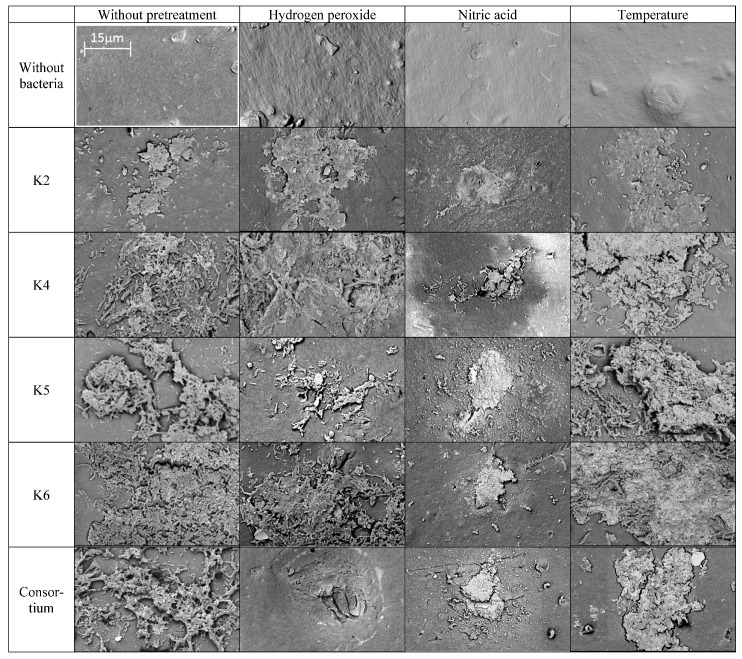
Scanning electron microscopy (SEM) micrographs of the biofilm formed on pretreated LDPE by bacterial strains isolated from commercial compost. Magnification 10,000×, voltage 5 kV, detector BSD. The scale bar is the same for all images.

The colonization potential of microorganisms was determined by comparing the images of the films exposed to bacteria with those of the control LDPE films. As illustrated in Figure 6, a substantial bacterial biofilm was observed on the polyethylene surface after a 180-day incubation period. The biofilm layer varied morphologically depending on the bacterial strain and the pretreatment of the film.

Further analysis of untreated and microbially treated polyethylene films was focused on investigating changes formed on the polymer surfaces. The surface of the abiotic sample was preserved intact. In contrast, the bacteria-treated films exhibited signs of degradation, including pitting, cracking, and rippling (Figure 6). A similar effect was observed by Devi et al. [62]. The employment of nitric acid as a pretreatment of LDPE caused erosion of the polymer surface and affected its colonization by isolated bacterial strains. It was found that this kind of pretreatment impeded bacterial adhesion to the LDPE, which did not form a dense biofilm with a well-developed EPS structure. Only separated spots were observed, where a more developed group of cells could be observed growing on the film surface.

### 3.3. Phytotoxicity

**Figure 7:**
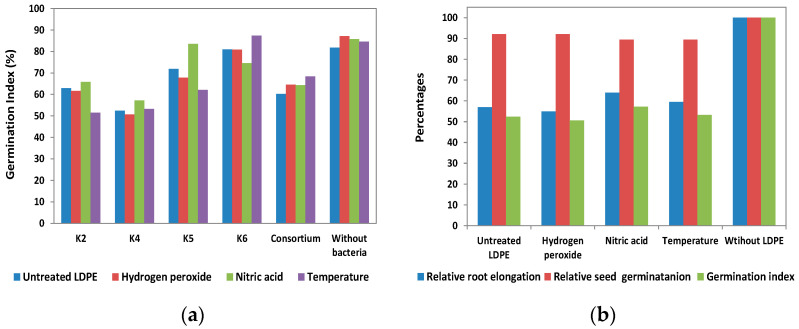
Phytotoxicity studies of LDPE biodegradation products. (**a**) Effect of cell-free effluent from the cultures on germination index; (**b**) the impact of compounds formed during the pretreated LDPE biodegradation by strain K4 on relative root elongation, relative seed germination, and germination index.

To estimate the effect of LDPE biodegradation products generated by selected strains on plants, phytotoxicity tests were performed, and the following variables were calculated: relative root elongation, relative seed germination, and germination index. Phytotoxicity assessment of metabolites formed during biodegradation was performed for experiments with virgin and pretreated plastic (Figure 7). From the results obtained, it can be concluded that the degradation products of LDPE induce a moderately toxic effect on the plants. The germination index for the control containing LDPE without bacterial inoculation is presented in Figure 7a. As illustrated in Figure 7a, the germination indices for the LDPE control without bacteria were generally higher than those obtained for LDPE subjected to pretreatment followed by bacterial incubation. The sole exception was observed for HNO_3_-pretreated LDPE incubated with strain K6, for which the degradation products did not exert an inhibitory effect on seed germination. Regardless of LDPE pretreatment, the most negative effect on germination index (seed germination) was shown by the leachate from the culture of the bacterial strain K4 (Figure 7a). As demonstrated in Figure 7b, relative root length exerts a more significant influence on the GI value than relative seed germination. During the LDPE biodegradation tests, this strain caused the greatest loss of polymer mass, probably resulting in the highest concentration of LDPE degradation products in the cultures. This, in turn, is likely to have increased the phytotoxicity of the effluents on plants. As demonstrated in Figure 7b, relative root length exerts a more significant influence on the GI value than relative seed germination.

The results of studies reported in the literature imply that products of LDPE biodegradation do not always demonstrate a toxic effect; indeed, in some cases, biostimulative effects on plants can even be observed. The degradation of LDPE using *Bacillus subtilis* and *Streptomyces labedae* did not induce any abnormalities in the germination of *Trigonella foenum* seeds. This observation indicates that these bacteria do not produce toxic byproducts during the decomposition of LDPE [78]. An increase in the relative seed germination and germination rate, as well as a shortening of the roots of Lactuca sativa L., was found after treatment with the effluent obtained from the culture of the *Stenotrophomonas* sp. and *Alcaligenaceae* [77]. Rani et al. observed a significantly higher level of germination of seeds exposed to *B. licheniformis* SARR1/LDPE culture effluents compared to the LDPE sample that was not subjected to bacterial treatment, so the use of *B. licheniformis* SARR1 for LDPE degradation even increased the environmental usefulness of the process [79].

Although microbial biodegradation of plastics is often regarded as environmentally safe, increasing evidence suggests that this assumption may not be entirely accurate. Several studies have reported the effects of microplastics on plant germination when introduced into the soil. Generally, such microplastics do not exhibit immediate inhibitory effects on plant growth, as they are relatively inert in the short term. However, during the biodegradation of polyethylene (PE) by selected microbial strains, we observed partial inhibition of wheat seed germination and root growth compared to PE not exposed to bacterial action, which is consistent with literature reports.

Previous studies have provided detailed analyses of LDPE biodegradation products generated by various microbial strains, demonstrating that individual compounds can inhibit germination and plant development in a concentration-dependent manner. Moreover, supernatants may also contain other inhibitory substances not directly associated with polyethylene degradation. In natural environments, such as open landfills, polyethylene undergoes photodegradation via the Norrish reaction, generating free radicals, terminal vinyl groups, ketones, and subsequently alcohols, carboxylic acids, aldehydes, and esters [80]. During microbial biodegradation, PE is first oxidized to alcohols by monooxygenases, which are then converted to aldehydes by alcohol dehydrogenases. Aldehydes are further oxidized to fatty acids by aldehyde dehydrogenases, and fatty acids are metabolized via β-oxidation to CO_2_ and energy [81]. GC–MS analyses have identified a wide range of compounds in PE biodegradation leachates, including phthalates, aromatic hydrocarbons, alkanes, and fatty acids. For example, degradation of polyethylene by Lysinibacillus fusiformis VASB14/WL and Bacillus cereus VASB1/TS yielded 1-trimethylsilylmethanol, hexadecanoic acid, 1,2,3,4-tetramethylbenzene, dibutyl phthalate, and other aromatic derivatives [82]. Similarly, Kyaw et al. [23] detected methyl tetrachloroethylene, benzene derivatives, long-chain alkanes, and esters during PE biodegradation. Mahalakshmi et al. [83] reported the presence of octadecadienoic acid, benzenedicarboxylic acid, and cyclopropanebutanoic acid after biodegradation by Bacillus and Pseudomonas strains.

Fungal degradation of polyethylene has also been linked to phytotoxic effects. Products obtained from Aspergillus terreus significantly reduced wheat germination and root growth, with elongation inhibition rates reaching 100% after three days [84]. Degradation products of Alternaria alternata were less toxic but still reduced germination and germination index values. GC–MS analysis identified Bis(2-ethylhexyl) phthalate as a major compound in these leachates [84]. Similarly, polyethylene degradation by A. terreus and A. sydowii reduced the germination index of sorghum to approximately 50% and produced a range of compounds including dibutyl phthalate, benzenediols, and fatty acids [85].

In our experiments, a marked decrease in the germination index of wheat seeds was observed, indicating the inhibitory effects of LDPE biodegradation by the isolated bacterial strains. These findings suggest that certain metabolites generated during the degradation process may be phytotoxic. Therefore, future studies should incorporate targeted and untargeted GC–MS analyses to identify the specific compounds responsible and to better understand the relationship between the chemical profile of biodegradation products and their phytotoxic effects on plants.

## 4. Conclusions

This study demonstrates that microorganisms isolated from compost containing polyethylene can degrade the polymer matrix, albeit at a slow rate. The compost-derived *Actinomycetes* caused measurable weight loss, surface oxidation, and morphological changes to polyethylene films, confirming microbial colonization as a key step in the degradation process. Acid pretreatment proved most effective, enhancing surface hydrophilicity, introducing oxidized functional groups, and producing minimal phytotoxic byproducts. These findings highlight the potential of targeted pretreatment to improve microbial access to polyethylene and point to the value of exploring longer and combined pretreatment strategies to address the growing challenge of plastic waste pollution.

## Figures and Tables

**Table 1:** Impact of compost-isolated *Actinomycetes* on physicochemical properties of pretreated LDPE.

K2	Untreated LDPE	LDPE(H_2_O_2_)	LDPE(HNO_3_)	LDPE(T)
Biodegradation [%]	1.9	0.8	2.7	2.4
Water contact angle change [%]	1.1	0.0	16.7	1.1
pH change [%]	3.8	3.3	2.4	3.6
**K4**	**Untreated LDPE**	**LDPE(H_2_O_2_)**	**LDPE(HNO_3_)**	**LDPE(T)**
Biodegradation [%]	3.2	1.8	4.6	4.1
Water contact angle change [%]	1.1	8.9	12.2	0.0
pH change [%]	5.9	5.2	5.3	5.2
**K5**	**Untreated LDPE**	**LDPE(H_2_O_2_)**	**LDPE(HNO_3_)**	**LDPE(T)**
Biodegradation [%]	0.9	3.0	2.3	2.5
Water contact angle change [%]	10.0	6.7	8.9	4.4
pH change [%]	3.5	4.5	4.5	4.5
**K6**	**Untreated LDPE**	**LDPE(H_2_O_2_)**	**LDPE(HNO_3_)**	**LDPE(T)**
Biodegradation [%]	2.0	1.7	2.2	2.5
Water contact angle change [%]	0.0	10.0	21.1	10.0
pH change [%]	6.2	3.8	3.3	3.6
**Consortium**	**Untreated LDPE**	**LDPE(H_2_O_2_)**	**LDPE(HNO_3_)**	**LDPE(T)**
Biodegradation [%]	2.1	1.6	4.3	2.9
Water contact angle change [%]	8.9	10.0	11.1	5.6
pH change [%]	3.3	1.8	2.7	2.6

## Data Availability

The original contributions presented in this study are included in the article. Further inquiries can be directed to the corresponding author.

## Supplementary Materials

The following supporting information can be downloaded at https://www.mdpi.com/article/10.3390/polym17172318/s1, Figure S1: Schematic diagram of the research workflow. Table S1: Average mass of LDPE (± SD) before and after degradation by isolated bacterial strains.; Table S2: Average Water Contact Angle of LDPE (± SD) before and after degradation by isolated bacterial strains.; Table S3: Average pH of the culture (± SD) before and after degradation by isolated bacterial strains.; Table S4: Effect of cell-free effluent from the cultures on wheat (*Triticum aestivum* L.) seeds after degradation by isolated bacterial strains. Average Relative root elongation (± SD).

## Abbreviations

The following abbreviations are used in this manuscript:

LDPE	Low-density polyethylene
LDPE(WP)	Low-density polyethylene without pretreatment
LDPE(H_2_O_2_)	Low-density polyethylene after hydrogen peroxide pretreatment
LDPE(HNO_3_)	Low-density polyethylene after nitric acid pretreatment
LDPE(T)	Low-density polyethylene after thermal pretreatment
ATR-FTIR	Attenuated total reflection–Fourier-transform infrared spectroscopy
OD	Optical density
CI	Carbonyl index
SEM	Scanning electron microscopy
WCA	Water contact angle
GI	Germination index
