# Supplementary material for: Potential of Compost-Derived Actinomycetes for Low-Density Polyethylene Degradation
Source: Polymers (Basel). 2025 Aug 27;17(17):2318. doi: 10.3390/polym17172318 (PMC12431224; doi:10.3390/polym17172318)
Supplement: Supplementary file 1 [file polymers-17-02318-s001.zip › polymers-3809479-supplementary.pdf]

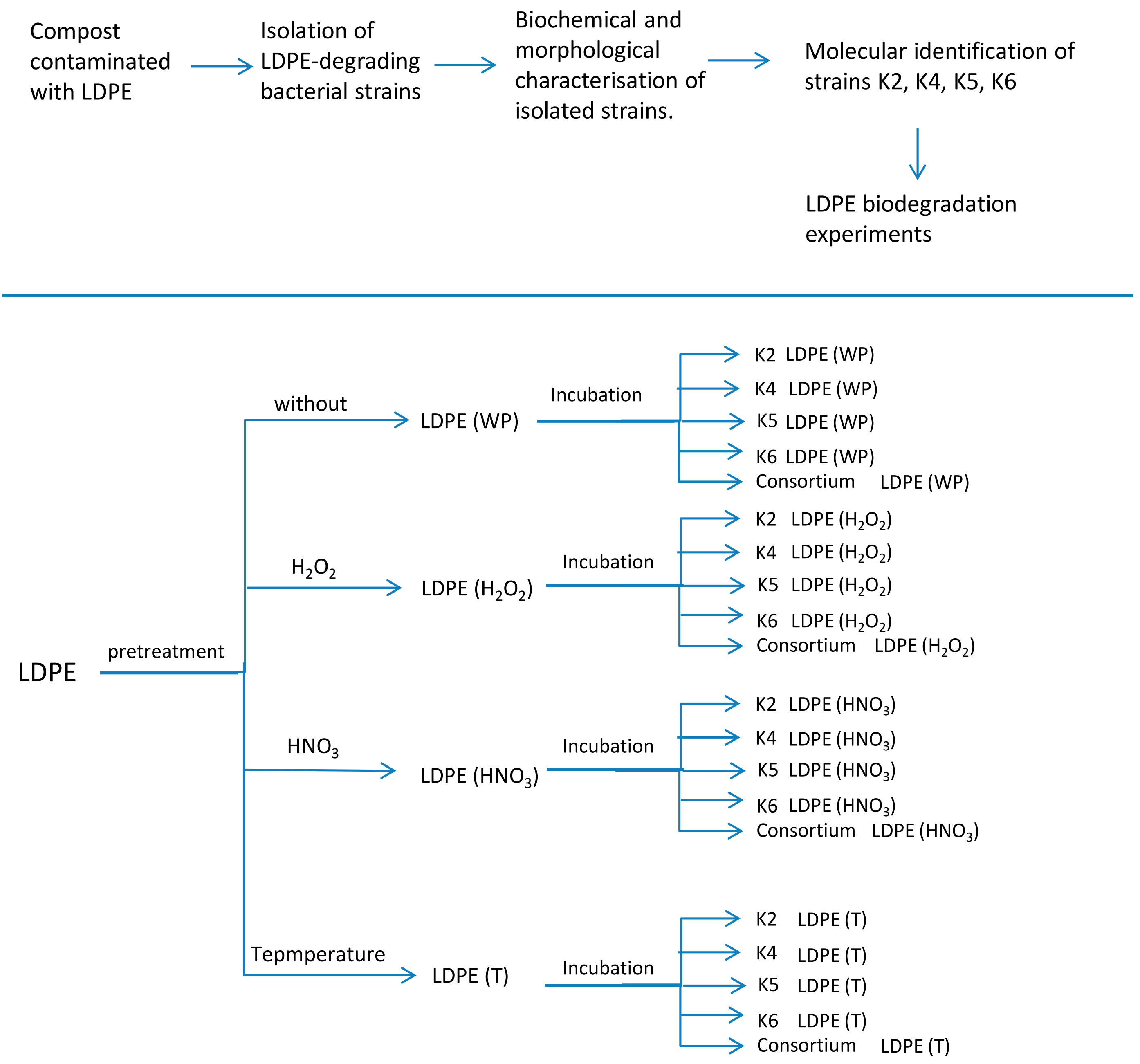

LDPE- low dencity ethylene

LDPE (WP)- LDPE without pretreatment

LDPE (H_2O_2)- LDPE after hydrogen peroxide pretreatment

LDPE (HNO_3)- LDPE after nitric acid pretreatment

LDPE (T) – LDPE after thermal pretreatment

Figure S1. Schematic diagram of the research workflow

Table S1. Average mass of LDPE (± SD) before and after degradation by isolated bacterial strains.

Initial weight										
	K2		K4		K5		K6		Consortium	
	mean	SD	mean	SD	mean	SD	mean	SD	mean	SD
LDPE(WP)	0.0860	0.0008	0.0912	0.0011	0.0970	0.0007	0.0863	0.0004	0.0958	0.0010
LDPE(H_2O_2)	0.1026	0.0012	0.0881	0.0009	0.0789	0.0010	0.0817	0.0013	0.0996	0.0010
LDPE(HNO_3)	0.0930	0.0008	0.0843	0.0011	0.0910	0.0011	0.0965	0.0005	0.0770	0.0011
LDPE(T)	0.0909	0.0008	0.0655	0.0005	0.0730	0.0003	0.0911	0.0008	0.0650	0.0009
Final weight										
LDPE(WP)	0.0844	0.0004	0.0883	0.0007	0.0961	0.0008	0.0846	0.0010	0.0938	0.0010
LDPE(H_2O_2)	0.1018	0.0009	0.0865	0.0011	0.0765	0.0004	0.0803	0.0007	0.0980	0.0008
LDPE(HNO_3)	0.0905	0.0004	0.0804	0.0009	0.0889	0.0005	0.0944	0.0006	0.0737	0.0007
LDPE(T)	0.0884	0.0004	0.0628	0.0009	0.0712	0.0007	0.0888	0.0010	0.0631	0.0006

Table S2. Average Water Contact Angle of LDPE (± SD) before and after degradation by isolated bacterial strains.

	K2		K4		K5		K6		Consortium		Initial	
	mean	SD	mean	SD	mean	SD	mean	SD	mean	SD	mean	SD
LDPE(WP)	89	2.05	89	1.70	81	1.41	90	0.47	82	1.41	90	0.94
LDPE(H_2O_2)	90	0.47	82	2.16	84	1.70	81	1.41	81	1.41	89	1.25
LDPE(HNO_3)	75	1.41	79	3.09	82	0.94	71	1.63	80	0.82	88	0.47
LDPE(T)	89	1.63	90	0.47	86	1.63	81	0.47	85	1.25	90	0.47

Table S3. Average pH of the culture (± SD) before and after degradation by isolated bacterial strains.

	K2		K4		K5		K6		Consortium		Initial	
	mean	SD	mean	SD	mean	SD	mean	SD	mean	SD	mean	SD
LDPE(WP)	6.35	0.02	6.21	0.02	6.37	0.02	6.19	0.02	6.38	0.00	6.60	0.02
LDPE(H_2O_2)	6.38	0.02	6.26	0.01	6.30	0.02	6.35	0.02	6.48	0.01	6.60	0.02
LDPE(HNO_3)	6.44	0.03	6.25	0.03	6.30	0.02	6.38	0.01	6.42	0.01	6.60	0.02
LDPE(T)	6.36	0.02	6.26	0.02	6.30	0.01	6.36	0.02	6.43	0.02	6.60	0.02

Table S4. Effect of cell-free effluent from the cultures on wheat (Triticum aestivum L.) seeds after degradation by isolated bacterial strains. Average Relative root elongation (± SD) .

	K2		K4		K5		K6		Consortium	
	mean	SD	mean	SD	mean	SD	mean	SD	mean	SD
LDPE(WP)	4.0	1.2	5.1	1.4	4.0	1.1	4.0	1.1	3.2	0.9
LDPE(H_2O_2)	3.5	1.0	4.9	1.6	3.7	1.0	4.5	1.2	3.4	1.0
LDPE(HNO_3)	3.7	0.9	5.7	1.8	4.7	1.2	4.0	1.1	3.5	0.7
LDPE(T)	3.4	1.0	5.3	2.1	3.7	1.2	4.5	1.0	3.6	0.8
water	5.4	1.4	9.0	2.1	5.3	1.4	5.0	1.3	4.8	1.1
